# A novel model for relation prediction in knowledge graphs exploiting semantic and structural feature integration

**DOI:** 10.1038/s41598-024-63279-2

**Published:** 2024-06-05

**Authors:** Jianliang Yang, Guoxuan Lu, Siyuan He, Qiuer Cao, Yuenan Liu

**Affiliations:** 1 School of Information Resource Management, Renmin University of China, Beijing, China; 2 School of Journalism and Communication, Renmin University of China, Beijing, China

**Keywords:** Knowledge graph, Relation prediction, BERT, Relation message passing, Computational science, Computer science, Information technology, Scientific data

## Abstract

Relation prediction is a critical task in knowledge graph completion and associated downstream tasks that rely on knowledge representation. Previous studies indicate that both structural features and semantic information are meaningful for predicting missing relations in knowledge graphs. This has led to the development of two types of methods: structure-based methods and semantics-based methods. Since these two approaches represent two distinct learning paradigms, it is difficult to fully utilize both sets of features within a single learning model, especially deep features. As a result, existing studies usually focus on only one type of feature. This leads to an insufficient representation of knowledge in current methods and makes them prone to overlooking certain patterns when predicting missing relations. In this study, we introduce a novel model, RP-ISS, which combines deep semantic and structural features for relation prediction. The RP-ISS model utilizes a two-part architecture, with the first component being a RoBERTa module that is responsible for extracting semantic features from entity nodes. The second part of the system employs an edge-based relational message-passing network designed to capture and interpret structural information within the data. To alleviate the computational burden of the message-passing network on the RoBERTa module during the sampling process, RP-ISS introduces a node embedding memory bank, which updates asynchronously to circumvent excessive computation. The model was assessed on three publicly accessible datasets (WN18RR, WN18, and FB15k-237), and the results revealed that RP-ISS surpasses all baseline methods across all evaluation metrics. Moreover, RP-ISS showcases robust performance in graph inductive learning.

## Introduction

Knowledge graphs are a critical advancement in the field of information science and computer science, embodying a structured representation of human knowledge. In an age marked by the rise of large language models such as GPT-4, knowledge graphs act as a complementary resource to these models, providing structured insights that enhance and deepen their semantic understanding^1,2^. These graphs find applications in diverse fields ranging from semantic search and question answering to intelligent decision-making^3–5^. However, the construction of robust knowledge graphs has been fraught with challenges, primarily due to their often incomplete and fragmented nature. For instance, about 71% of the entities in Freebase lack data regarding their place of birth^6^, and DBpedia lacks descriptions for over 66% of scientists^7^. These substantial gaps in data necessitate a focused effort towards knowledge graph completion (KGC), which aims to enrich the graph by addressing missing data, thereby enhancing the comprehensiveness and robustness of the complex structure^8,9^.

Both structural features and semantic features are meaningful for relation prediction. For example, based on structural information, we can infer a new ’Grandfather_of’ relation from two consecutive ‘Father_of’ relations. From the perspective of semantic information, we can deduce a corresponding ‘Child_of’ relation from a ‘Mother_of’ relation. However, these two types of features represent two different learning paradigms: structure-based methods and semantics-based methods. Structure-based methods like TransE^10^, TransR^11^ and R-GCN^12^, treat entities and relations as nodes and edges, using structure patterns to learn representations of nodes and edges to predict missing relations. On the other hand, semantics-based methods like KG-BERT^13^, treat entities and edges as text, transforming the problem of relation prediction into one of sentence classification. Since these two approaches are completely different learning paradigms, it is difficult to fully utilize both types of features within a single learning model, especially deep features^14^. These issues prevent fully leveraging the combination of structural and semantic features, which is crucial for effective prediction. Thus, there's a need for methods that can more skillfully utilize both structural and semantic aspects.

Our research addresses the challenge of effectively integrating structural and semantic information in knowledge graph completion, a problem inadequately solved by existing methods. This led to the creation of RP-ISS (Relation Predictor Integrating Semantic and Structural Features), a novel model designed for balanced and deep integration of both modalities. The RP-ISS was inspired by the Lin et al.^15^. It first dynamically initializes the representation of each entity using a BERT-style model. Then, the representations of entities are iteratively updated through an edge-based message-passing network. At the same time, edge representations can be synchronously updated according to entity representations. During initialization, semantic features are utilized for learning entity representations. In the iterative updating process, graph structural patterns are used for learning entity and edge representations. In this way, edge representations contain not only deep semantic features but also deep structural features, effectively integrating text semantic encoding with structural information. This approach, which contrasts with the partial integrations of previous efforts, has proven successful in experiments using standard datasets like WN18RR, WN18, and FB15k-237. RP-ISS's performance confirms the effectiveness of our method, advancing knowledge graph completion and opening new research directions.

The key contributions of this study are:

- A novel model is proposed to address the challenge of integrating semantic and structural features in relation prediction in knowledge graphs. The proposed model leverages text semantic information in the knowledge graph and extracts semantic features of entity nodes using RoBERTa.
- By incorporating semantic encoding, this study introduces structural information and presents a novel edge-based relational message-passing network. This network enhances the transmission of edge messages through entity nodes.
- This study proposes a node embedding memory bank to mitigate the substantial computational pressure that the edge-based relational message-passing network imposes on the RoBERTa during sampling. The asynchronous update of the memory bank enables the effective integration of structural and semantic features, resulting in improved prediction performance without a significant increase in computational complexity.

## Related work and background

### KGC based on graph embedding

Relation prediction in knowledge graphs is a challenging task that involves predicting missing relations among richly semantically annotated nodes and multiple relation types. Early research in relation prediction predominantly employed knowledge embedding based on translational distance, such as TransE^10^ and TransR^11^. These methods utilize only a limited amount of structural information surrounding the given entities. Graph embeddings have gained popularity for their ability to analyze graph structural data. For representing knowledge graph nodes, Schlichtkrull et al.^12^ proposed relational graph convolution network (R-GCN). R-GCN is an encoder-decoder model for knowledge graph completion, in which different weight matrices are constructed based on diverse relations to aggregate various neighbor nodes. R-GCN is a classic method for knowledge graph completion using graph neural networks, with many subsequent approaches being proposed based on this model. Nathani et al.^16^ proposed KBGAT, which defines an attention layer and uses a hierarchical iteration to spread attention based on the concatenation of entity and relation embeddings. Lee et al.^17^ proposed a link prediction method based on relation paths. This method transforms the graph structure into path sequences using a path ranking algorithm and predicts missing entities and relations through sequence models. Peng et al.^18^ proposed KPE-PTransE, a relation prediction method for knowledge graph reasoning tasks based on relational paths and K-nearest neighbors. This method incorporates relational paths as important features, in addition to structural information. Ma et al.^19^ proposed a model based on graph attention faded mechanism, which enhances the representation ability of structural features by capturing multi-hop neighbor information.

### KGC based on pre-trained language model

Although graph embedding approaches have achieved impressive performance in relation prediction by representing nodes based on structural features, they tend to overlook the rich and latent semantic features of entities. To utilize these semantic features, Yao et al.^13^ proposed KG-BERT, which employs BERT for knowledge graph completion. BERT, pretrained on a large-scale corpus, possesses a strong semantic understanding and abundant implicit knowledge of natural language, allowing it to represent entities based on semantic features. This method obtains entity representations through entity names and descriptions, adapting input to fit the form of knowledge graph triples by using description sentences to represent entities. However, this approach has the limitation of only learning semantic features and implicit knowledge while disregarding structural information. Building on KG-BERT, Wang et al.^20^ proposed StAR to address the problem of link prediction. This model optimizes the input of KG-BERT by incorporating head entities and relations as inputs and trains the model using negative sampling. Due to the inclusion of relation information, the model achieves improved results in link prediction tasks but remains limited to link prediction applications. Nassiri et al.^21^ introduced the GilBERT model, a sophisticated approach grounded in the Transformer architecture for triple networks. The model creates an embedding space that aggregates information related to entities or relations within the knowledge graph. GilBERT generates textual sequences from factual data and further fine-tunes the pre-trained Transformer-based language model specifically for the triple network. This approach further translates the problem of knowledge graph completion into a spatial semantic search process, providing a novel perspective for knowledge graph completion based on textual semantic information. Nadkarni et al.^22^ conducted a systematic investigation into the integration of graph embedding with pre-trained language models for the purpose of knowledge graph completion. They introduced a series of fusion strategies, exemplified by KGE-BERT, which have been applied specifically to the task of medical knowledge graph completion. Fundamentally, this approach amalgamates shallow structural features with profound textual characteristics, thereby achieving a more nuanced and comprehensive completion of the knowledge graph. The synthesis of these distinct facets offers a robust and innovative methodology, underscoring the potential of multi-modal integration within the realm of knowledge representation. Shen et al.^23^ designed a novel method LASS that fuses semantic and structural features. To obtain semantic features, they adopted the KG-BERT approach, using pretrained language models to extract the semantic feature of entities. Instead of directly extracting structural features for structural information, their method employs a TransE-based scoring function to compute the probability scores of triples.

## Model design

### Problem definition

In a knowledge graph $\mathcal{G}=\left(\mathcal{V},\mathcal{E}\right)$, where $\mathcal{V}$ is a set of nodes and $\mathcal{E}$ is a set of edges, the graph is composed of multiple triples $\left\{h,r,t\right\}$, where $h$ and $t$ denote the head and tail nodes, and $r$ represents the relation. A representative task within the domain of knowledge graph completion is Link Prediction, which aims to predict the missing components of a triplet $\left\{h,r,t\right\}$. Some studies further subdivide Link Prediction into several subtasks^24,25^. In our work, the relation prediction problem that we have conducted can be defined as: predicting the missing relation based on the head and tail entities, symbolized as predicting the missing relation in $\left\{h,?,t\right\}$^26,27^.

Generally speaking, the fundamental concept of knowledge graph completion is to establish a scoring function $S\left(\cdot \right)$, which assigns a score to any given triplet $\left\{h,?,t\right\}$. If this triplet is present within the knowledge graph, it receives a high score, and vice versa. Building upon this idea, for the task of relation prediction, we can transform the scoring function into:

1 $$S\left(r\right)=\Phi (r;h,t;\mathcal{G})$$

where $r$, $h$ and $t$ respectively represent the relation, head entity, and tail entity. $\mathcal{G}$ denotes the knowledge graph where the triplets reside. $\Phi$ represents the method for calculating relation scores, used to compute the likelihood of the existence of relation r between h and t.

### Model architecture

**Figure 1:**
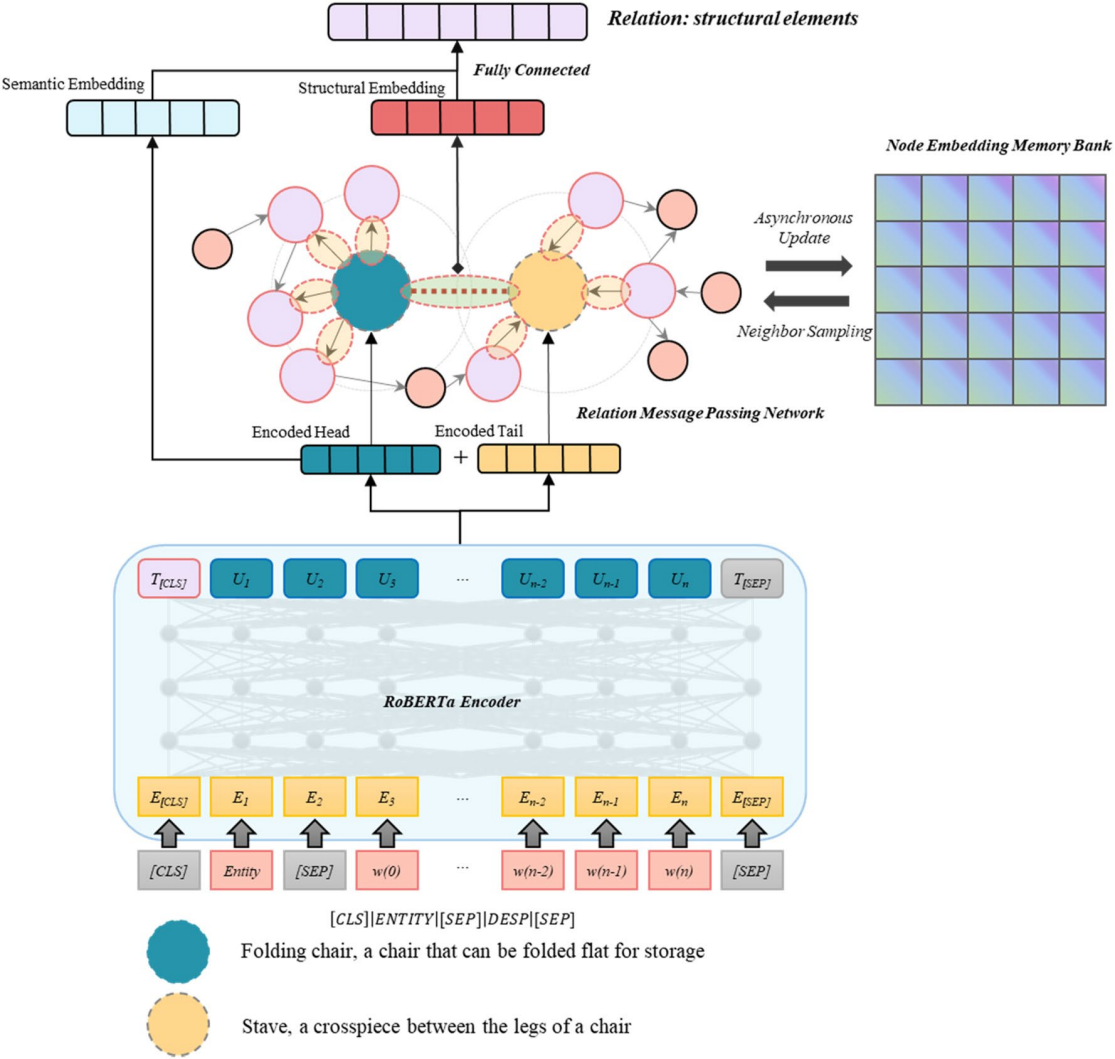
The architecture of our model RP-ISS.

To improve the prediction of missing relations by integrating textual semantic features and graph structural features, we propose the RP-ISS model. The model utilizes the tacit knowledge and semantic feature extraction ability of RoBERTa. Due to some advanced models for knowledge graph completion, such as KG-BERT and GilBERT, being end-to-end prediction models that cannot be used as PLMs, we primarily utilized RoBERTa in RP-ISS. To enable the model to process graph structural information, we design and apply a Relation Message Passing Network (as described in Section "Relational message passing"). The structure of RP-ISS is shown in Fig. 1.

To extract semantic feature of entities, we transform the information of an entity node into a set of tokens to serve as input for RoBERTa. For an entity, we transform its name and description into a token sequence in the form of [*CLS*][*Entity Name*][*SEP*][*Entity Description*]. Here, [*Entity Name*] represents the tokens of the entity's name. [*Entity Description*] represents the tokens of the entity's description. [*CLS*] is a special classification token. [*SEP*] signifies the separation of sentences. This set of tokens consists of two sentences, ${S}_{e}$ and ${S}_{d}$. Sentence ${S}_{e}$ contains the tokens of the entity to be encoded, and sentence ${S}_{d}$ contains the tokens of its description. To predict the relations between the two nodes, we construct the tokens of head entity $h$ and tail entity $t$ separately, and input them into the same RoBERTa for encoding. The encoded output of $h$ and $t$ from the RoBERTa are represented as $u$ and $v$ respectively.

To extract the structural feature of the graph, we put the encoded vectors $u$ and $v$ from RoBERTa through a Relation Message Passing Network (as described in Section "Relational message passing"). In the Relation Message Passing Network, we use subgraph sampling to recursively sample and aggregate the information of neighboring edges related to the current edge, to obtain the representation of the current edge. The edge representation is calculated based on the entity embeddings with a fully connected layer. During each sampling hop, multiple nodes are sampled and edge representations are calculated. The vectors of these sampled nodes are from the previous round of RoBERTa encoding stored in the Node Embedding Memory Bank and do not participate in the current backpropagation computation. The encoding process can be expressed as follows:

2 $${r}_{sema}=\sigma \left(\left[u,v\right]\cdot W+b\right)$$

3 $${r}_{strc}^{l}=\sigma \left(\left[{r}_{h,t},{s}_{{e}_{h}}^{l-1},{s}_{{e}_{t}}^{l-1}\right]\cdot {W}^{l}+{b}^{l}\right) ,{e}_{h}\in E\left(h\right),{e}_{t}\in E\left(t\right)$$

where ${r}_{sema}$ represents the semantic representation of the edge between an entity pair and while ${r}_{strc}^{l}$ refers to the structural representation of the edge between the entity pair. ${r}_{h,t}$ denotes the initial representation of the edge between head entity $h$ and tail entity $t$. ${s}_{e}^{l-1}$ represents the result of sampling a subgraph and aggregating structural information, which aggregates information from the edges connected to the head node ${e}_{h}$ and the edges connected to the tail node separately ${e}_{t}$. $l$ represents the number of hops of the sampled subgraph, also known as sampling depth.

Our goal is to predict the type of relation that connecting $h$ and $t$, that is, to find the most appropriate $r$ for the incomplete triple $\left\{h,?,t\right\}$. Once we calculated the semantic representation vector ${r}_{sema}$ and the structural feature representation vector ${r}_{strc}^{l}$, we concatenate ${r}_{sema}$ and ${r}_{strc}^{l}$ into a comprehensive representation ${e}_{r}$ Then we pass ${e}_{r}$ through a fully connected layer to adjust the dimensions and output a probability distribution representing the relation type of ${e}_{r}$. The softmax function for classification activates the final output.

### Relational message passing

Our study explores integrating RoBERTa's linguistic capabilities with GNN's structural insights to improve relation prediction. A simple stacking of RoBERTa and GNN creates excessive computational load due to GNN's need to aggregate features from surrounding nodes. We propose a novel method combining RoBERTa's strengths with knowledge graph structures through an edge-based relational message-passing mechanism and a node embedding memory bank. This allows seamless integration of RoBERTa's encoded entity output into the message-passing network, enhancing performance without the computational burden of a stacked model.

#### Edge-based relational message passing

**Figure 2:**
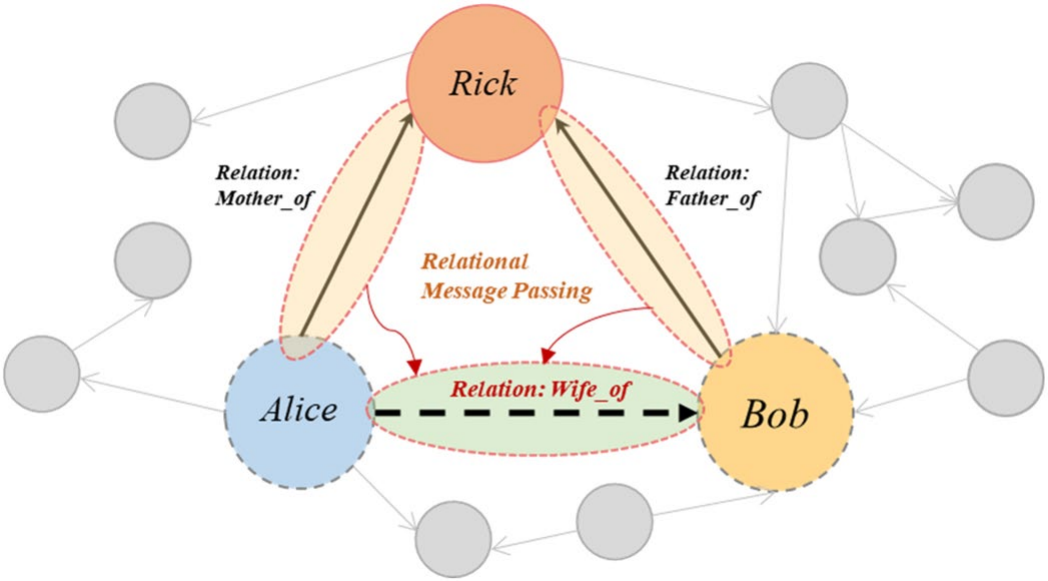
Illustration of Edge-based relational message passing.

The goal of the relation prediction task is to predict the type of the relation between two nodes. Therefore, we need to focus on the representation of the edges, rather than the representation of the nodes, which is the focus of most GNN models. Thus, we propose an edge-based relational message passing method. As shown in Fig. 2, we provide an example to illustrate how the edge-based relational message passing network helps us predict missing relations. Suppose there is a missing relation between the entities Alice and Bob, with the relationship type being Wife_of. This means that Alice is Bob's wife. There exists another entity Rick, whose mother and father are Alice and Bob respectively. When predicting this relation, the Edge-based relational message passing network can encode the information of the relation between Alice and Rick as well as between Bob and Rick, and pass it onto the current edge awaiting prediction. Compared to the general node-based message passing method, this method treats edges as nodes for message passing and information aggregation. The information aggregated on an edge is derived from the edges connected to the two entities to which the edge is linked. Nonetheless, considering the potentially large number of edges present in a graph, in our method, we do not directly store the representations of edges, but calculate them through the nodes. The message passing process of edges can be expressed as:

4 $${e}_{h,t}=\sigma \left(\left[{n}_{h},{n}_{t}\right]\cdot W+b\right)$$

5 $${m}_{h}=aggr\left(\left\{{e}_{h,\text{i}}\right\}\right),i\in N\left(h\right),i\ne t$$

6 $${m}_{t}=aggr\left(\left\{{e}_{j,\text{t}}\right\}\right),j\in N\left(t\right),j\ne h$$

7 $${e}_{h,t}{\prime}= U\left(\left[{e}_{h,t},{m}_{h},{m}_{t}\right]\right)$$

where ${e}_{h,t}$ is the representation of the edge between an entity pair, which is calculated based on the head ${n}_{h}$ and tail ${n}_{t}$. We use a fully connected layer to compute the representation of the edge ${e}_{h,t}$. ${m}_{h}$ is the message from all edges connected to the head entity except for the edge ${e}_{h,t}$. ${m}_{t}$ is the message from representation of all edges connected to the tail entity except for the edge ${e}_{h,t}$. $N\left(h\right)$ and $N\left(t\right)$ are the neighbor nodes of entity $h$ and $t$. ${m}_{h}$ and ${m}_{t}$ are calculated through an aggregate function. ${e}_{h,t}{\prime}$ is the updated representation of ${e}_{h,t}$, which is calculated by the original representation ${e}_{h,t}$, the messages from neighbor edges ${m}_{h}$ and ${m}_{t}$.$U$ represents the updating function for edge representations. Within $U$, ${e}_{h,t}$, ${m}_{h}$ and ${m}_{t}$ are concatenated and passed through a residual connection targeting ${e}_{h,t}$ to update its representation.

#### Node embedding memory bank

**Figure 3:**
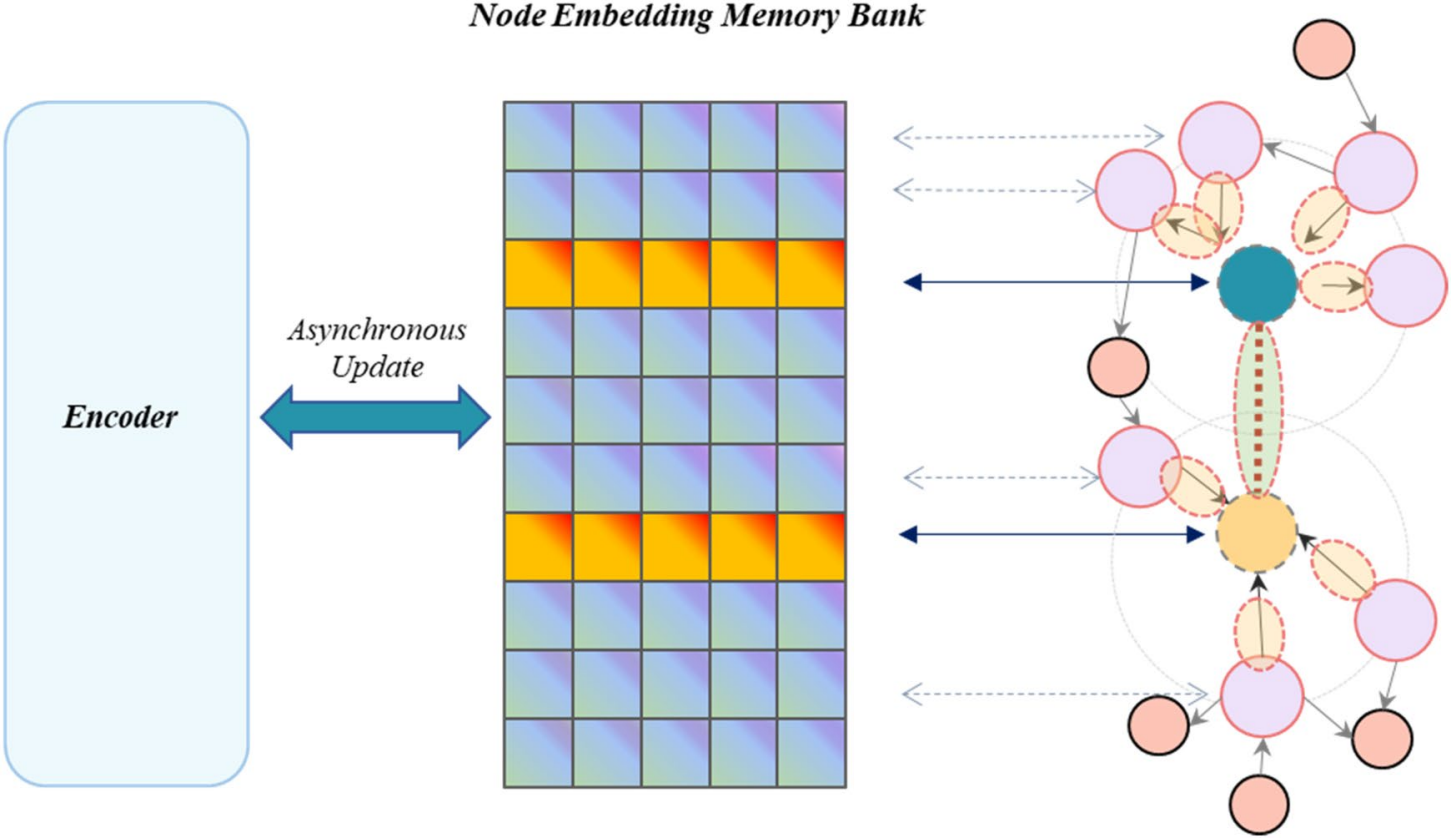
Illustration of node embedding memory bank.

Directly stacking RoBERTa with edge-based relation message passing networks incurs high computational costs due to the need for RoBERTa to encode each sampled neighboring node, exponentially increasing with sampling depth. To reduce this, especially during backward propagation in training, we implemented a node embedding memory bank (illustrated in Fig. 3). This bank stores RoBERTa-encoded nodes from previous training, updating all node pair embeddings after each training epoch. While sampling, it uses previously stored encodings, updating the memory bank with new encodings and propagating only the current node pair's gradient information to RoBERTa. However, this creates an inconsistency between the memory bank's node embeddings and RoBERTa's encodings, which we address with a weighted updating strategy for encoding results. This process can be represented as:

8 $${n}_{i}{\prime}=\lambda {s}_{i}+(1-\lambda ){n}_{i}$$

where ${n}_{i}{\prime}$ is the updated embedding of node $i$, ${s}_{i}$ represents the embedding of node $i$ encoded by BERT, ${n}_{i}$ refers to the original node embedding in the memory bank, and $\lambda$ is a weighting factor employed to regulate the ratio of the new encoding to the original node embedding. In our model, we set the value of the weighting factor $\lambda$ to 0.5.

## Experiments

### Datasets and baselines

We carried out experiments on three representative and publicly accessible datasets: WN18, WN18RR, and FB15k-237. WN18 is a knowledge graph that contains concepts and semantic relations of English words, based on WordNet. WN18RR is a subset of WN18 that has removed the reverse relations. FB15k-237, on the other hand, is a knowledge graph based on a large-scale general knowledge from Freebase with reverse relations also removed. The statistics of these three datasets are presented in Table 1.

**Table 1:** The statistics for the datasets.

	Train set	Validation set	Test set	Entities	Relations	Average degree
WN18RR	86,835	3034	3134	40,943	11	4.2
WN18	141,442	5000	5000	40,943	18	6.9
FB15k-237	272,115	17,535	20,466	14,541	237	37.4

To evaluate the performance of our proposed model, we compare it with several baseline methods in order to gain insights into its relative effectiveness: including TransE^10^, DistMult^28^, RotatE^29^, SimplE^30^, ComplEx^31^, R-GCN^12^, KG-BERT^32^,KPE-PTransE^18^, KGE-BERT(with routers)^22^, GilBERT^21^, LASS^23^. TransE, DistMult, RotatE, SimplE, ComplEx, and KPE-PTransE are very classic methods of knowledge representation, primarily expressing the structural features of knowledge graphs. R-GCN stands as a highly representative model for relation prediction, utilizing graph structure effectively. Conversely, KG-BERT, KGE-BERT and GilBERT emerges as an equally prominent model in this domain, leveraging pre-trained language models for relation prediction. LASS is one of the state-of-the-art models for knowledge graph completion based on semantic and structural features. We have used it to carry out relation prediction tasks, and it serves as one of our baselines.

We used the DGL-KE toolkit (see https://github.com/awslabs/dgl-ke/) to reproduce the results of the TransE, DistMult, RotatE, and ComplEx methods on the experimental dataset, and the results of SimplE, R-GCN, KG-BERT, KGE-BERT, GilBERT and LASS were reproduced using the accompanying source code from the original paper. The results of KPE-PTransE is based on the original paper.

### Experiments setting

Relation prediction involves predicting the missing relation between head and tail nodes, i.e., predicting the missing part in $\left\{h,?,t\right\}$. We use a multi-class classification model to predict the missing relation. Analogous to the evaluation method employed in link prediction, we utilize the probability distribution generated by the model to compute MRR, Hits@1, and Hits@3. These metrics serve as indicators of the model's performance.

Our model was implemented in PyTorch, using RoBERTa for node sequence encoding and the Adam optimizer for training. We determined the optimal learning rates to be 5e−5 for RoBERTa and 3e−4 for the Relation Message Passing Network through grid search. A warmup mechanism was applied, with warmup steps set at 10% of total training steps, and training capped at 30 epochs to avoid fast convergence of RoBERTa's final layers.To save training time and prevent overfitting, we employ early stopping. Batch sizes were set at 1000 for WN18 and WN18RR, and 500 for FB15k-237, with a maximum input sequence length of 50. We used mixed precision to save GPU memory and expedite training. The models were trained and tested on 4 Nvidia A5000 RTX GPUs.

## Results and discussion

### Main results

We recorded and compared the experimental outcomes of our proposed model and the established baseline models. The main results are shown in Table 2. RP-ISS-Mean and RP-ISS-Attn are two different methods for aggregating information from neighboring edges in a graph-based relation message passing network. RP-ISS-Mean aggregates information from neighboring edges through mean pooling, while RP-ISS-Attn uses a multi-head attention mechanism to aggregate information from neighboring edges.

**Table 2:** The main results on WN18RR, WN18 and FB15K-237 in relation prediction task.

	WN18RR MRR Hits@1 Hits@3			WN18 MRR Hits@1 Hits@3			FB15K-237 MRR Hits@1 Hits@3		
TransE	78.26	67.13	87.47	97.11	95.41	97.93	97.02	94.86	98.16
DistMult	84.83	78.82	88.85	78.77	58.51	98.98	87.22	80.49	93.40
RotatE	80.01	73.27	81.94	97.99	98.13	99.04	97.17	94.65	97.56
SimplE	73.46	66.22	75.60	97.47	96.75	97.53	96.70	95.65	98.46
ComplEx	82.48	76.92	85.16	98.09	97.15	98.17	92.41	87.80	96.50
R-GCN	82.19	74.95	84.79	90.90	82.38	97.90	93.14	90.31	94.19
KG-BERT	94.23	91.72	96.77	96.67	94.62	98.16	97.30	95.21	98.61
KPE-PTransE	85.30	87.50	94.70	98.70	98.20	98.90	96.20	**96.00**	98.50
GilBERT	94.15	91.65	96.72	96.54	94.71	98.06	95.11	91.40	96.98
KGE-BERT	94.79	92.41	96.12	96.45	95.68	97.93	97.22	94.28	98.96
LASS	95.89	94.23	96.42	96.67	95.89	97.97	97.35	95.49	98.78
RP-ISS-Mean	97.80	95.83	99.21	98.40	97.20	99.47	97.40	94.71	99.31
RP-ISS-Attn	**98.91**	**97.93**	**99.97**	**99.33**	**98.82**	**99.90**	**97.78**	95.98	**99.61**

*The bold font indicates the best result among all outcomes.

As shown in Table 2, the RP-ISS-Attn model exhibits the best performance and outperforms RP-ISS-Mean. Additionally, the RP-ISS-Attn model significantly outperforms all the baseline models. Compared to the structure-based models (TransE, DistMult, RotatE, SimplE, ComplEx, KPE-PTransE, R-GCN), our model shows an average improvement of 5.7%, 4.2%, and 2.5% in MRR, Hits@1, and Hits@3, respectively, compared to the best performance of these models. Compared to the semantic-based models (KG-BERT, GilBERT and KGE-BERT), our model shows an average improvement of 2.7%, 4.0%, and 2.0% in MRR, Hits@1, and Hits@3, respectively, compared to the best performance of these models.

**Figure 4:**
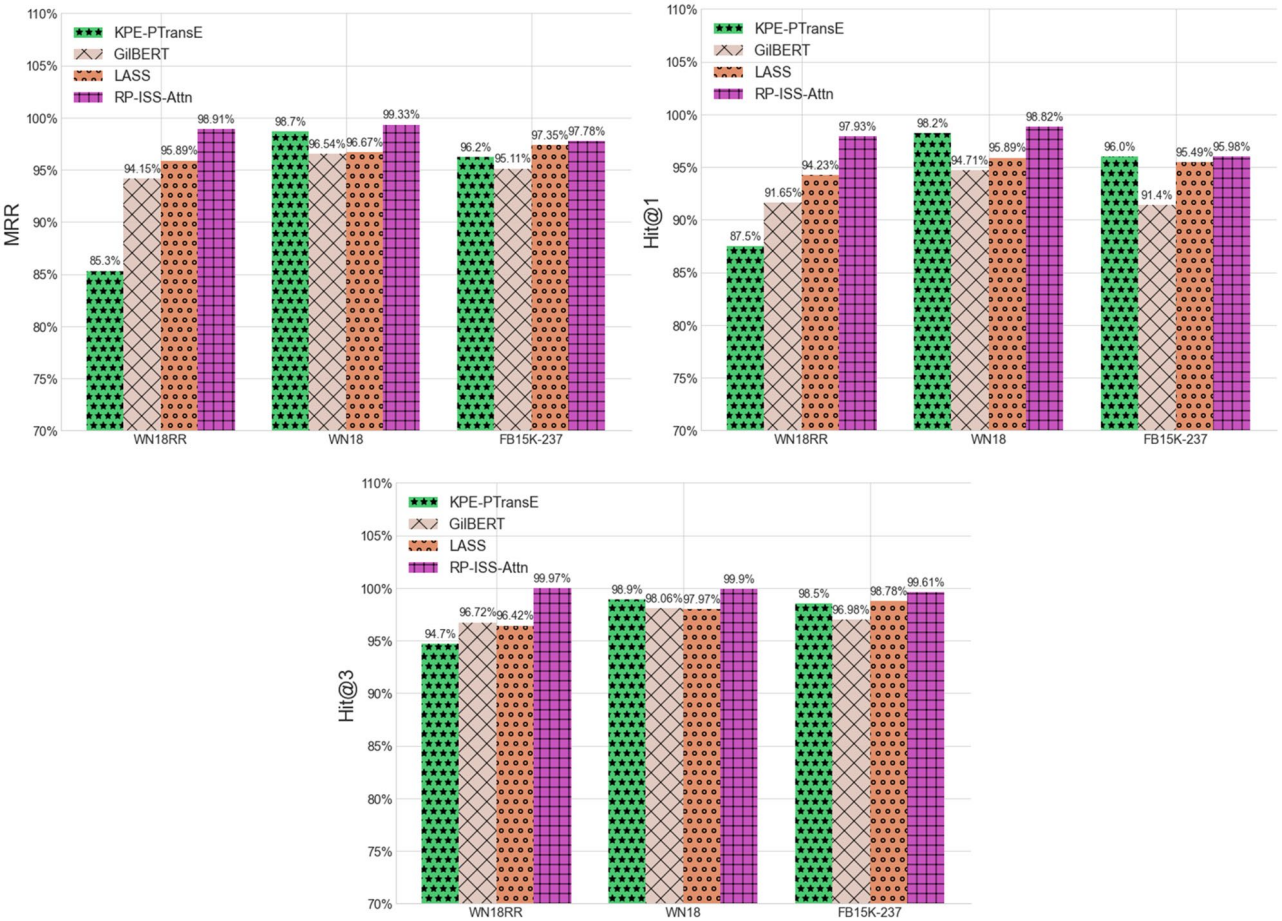
Comparison of RP-ISS with representative SOTA models.

As depicted in Fig. 4, our proposed RP-ISS model, adept at synthesizing both structural and semantic features, demonstrates notable advancements over established baseline models within the experimental datasets. We benchmarked against three salient models: KPE-TransE, grounded in graph embedding; GilBERT, which predominantly relies on textual semantic information; and LASS, which adeptly amalgamates both semantic and structural attributes. Each of these models epitomizes cutting-edge techniques in their respective domains. Upon analyzing the outcomes across the datasets, the RP-ISS model consistently outshines its counterparts. Specifically, it exceeds the KPE-TransE's performance by averages of 6.1% in MRR, 4.2% in Hits@1, and 2.6% in Hits@3. Against the GilBERT model, RP-ISS manifests superiority with average margins of 3.6% in MRR, 5.4% in Hits@1, and 2.6% in Hits@3. Furthermore, when juxtaposed with the LASS model, RP-ISS achieves a lead of 2.1% in MRR, 2.5% in Hits@1, and 2.2% in Hits@3.

The reason why RP-ISS can demonstrate a significant improvement over the baseline models is that it fully leverages structural and semantic features to predict missing relations. For those relations predicted through structural information, RP-ISS's edge-based message passing network module can effectively learn and represent these structural patterns, making accurate predictions for these relations. Therefore, compared to models primarily based on semantic features such as KG-BERT, GilBERT, KGE-BERT, RP-ISS shows a notable performance boost due to its utilization of structural features. For those relations that require inference from semantic information, RP-ISS's RoBERTa module can effectively learn and represent these semantic features and make correct predictions about these relations. Hence, compared to models mainly based on structural features like R-GCN, ComplEx, KPE-PTransE, RP-ISS also exhibits significant improvements. Since RP-ISS uses deep features relative to advanced models like LASS which also utilize both semantic and structural characteristics; it achieves a clear improvement as well.

### Ablation study

We conducted an ablation study to further understand the role of semantic feature and structural feature. As shown in Table 4. The results show that the performance of both models is significantly weaker than that of the RP-ISS model. Based on experiments on three datasets, the model without semantic feature fusion (RP-STRC) experienced a 2.87% decrease in MRR. The model without structural feature fusion (RP-SEM) saw a 2.94% MRR decrease.

On WN18RR, RP-STRC and RP-SEM had MRR reductions of 6.25% and 5.25%, Hits@1 reductions of 10.63% and 9.04%, and Hits@3 reductions of 2.17% and 1.75%, respectively. On WN18, RP-STRC and RP-SEM experienced MRR decreases of 1.21% and 2.80%, Hits@1 decreases of 2.04% and 5.42%, and Hits@3 decreases of 0.54% and 0.14%, respectively. On FB15K-237, RP-STRC and RP-SEM had MRR reductions of 1.16% and 0.79%, Hits@1 reductions of 1.73% and 1.67%, and Hits@3 reductions of 0.68% and 0.67%, respectively.

**Figure 5:**
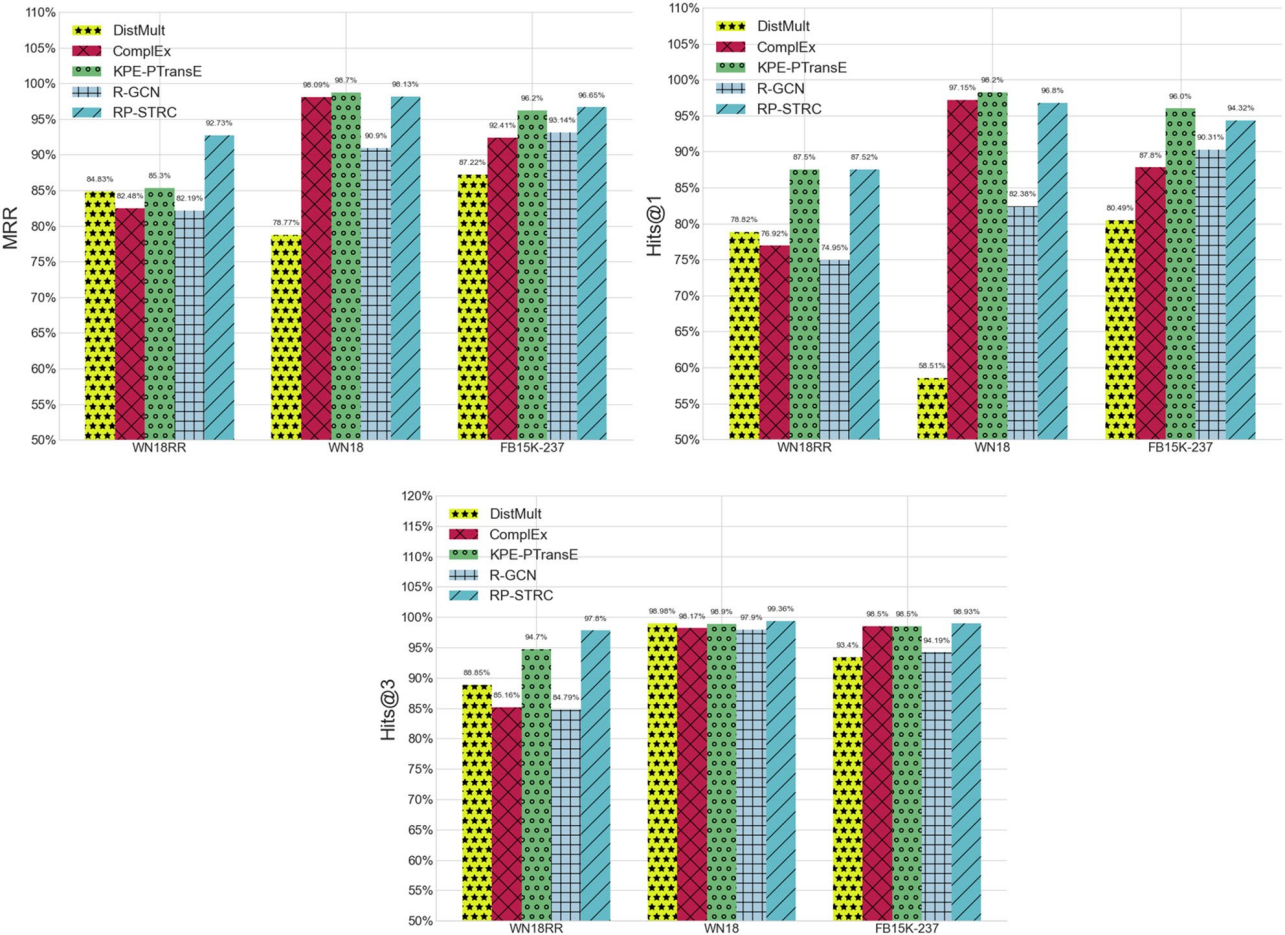
Comparison of RP-STRC with other structure-based methods.

RP-STRC exclusively utilizes the edge-based message passing network from our research, effectively capturing structural features. RP-STRC adopts randomly initialized embeddings. Specifically, we initialize the node embeddings in RP-STRC with a uniform distribution ranging from [− 1, 1]. During the learning process of RP-STRC, the node embeddings are iteratively updated. Comparisons with established structure-based approaches, as shown in Fig. 5, demonstrate RP-STRC's competitive performance in relation prediction tasks. Notably, RP-STRC, focused on structural features, marginally outperforms the semantic feature-based RP-SEM model (see Table 3), highlighting the importance of structural information in knowledge graph relation predictions.

**Table 3:** Results of ablation study on test set.

	WN18RR			WN18			FB15K-237		
	MRR	Hits@1	Hits@3	MRR	Hits@1	Hits@3	MRR	Hits@1	Hits@3
RP-ISS	98.91	97.93	99.97	99.33	98.82	99.90	97.78	95.98	99.61
RP-STRC (w/o semantic feat.)	92.73	87.52	97.80	98.13	96.80	99.36	96.65	94.32	98.93
	− 6.25%	− 10.63%	− 2.17%	− 1.21%	− 2.04%	− 0.54%	− 1.16%	− 1.73%	− 0.68%
RP-SEM (w/o structural feat.)	93.72	89.08	98.22	96.55	93.46	99.76	97.01	94.38	98.94
	− 5.25%	− 9.04%	− 1.75%	− 2.80%	− 5.42%	− 0.14%	− 0.79%	− 1.67%	− 0.67%

The RP-ISS model uses a hyperparameter λ to balance the weight of structural embeddings and semantic embeddings in the Node Embedding Memory Bank. To better study the impact of different settings of the hyperparameter λ on the model, we conducted experiments with various values of λ. As shown in Table 4, we found that setting the hyperparameter λ to 0.5 yields the best results. Looking at the distribution of results, it is evident that increasing λ significantly reduces model performance. A larger value for hyperparameter λ means fewer structural features and more semantic features are considered. This is consistent with conclusions drawn from ablation studies, which suggest that structural features are more important for relation prediction.

**Table 4:** Performance of the Model on the WN18RR with Different Hyperparameter λ Settings.

$\lambda$	$\lambda$=0.1	$\lambda$=0.3	$\lambda$=0.5	$\lambda$=0.7	$\lambda$=0.9
MRR	98.83	98.90	98.91	98.82	96.70

### Inductive prediction

We conducted an inductive graph learning experiment to evaluate our model's performance in inductive prediction, crucial in knowledge graph research. In real-world applications, domain knowledge graphs often expand over time, requiring models to handle different graph structures during training and prediction (inductive tasks)^33^. Our approach uses BERT to convert node information into token sequences, aiding inductive learning. To assess this, we performed experiments on the WN18RR dataset by simulating an inductive task, varying the number of nodes in the training set and training models with different reduction ratios.

**Figure 6:**
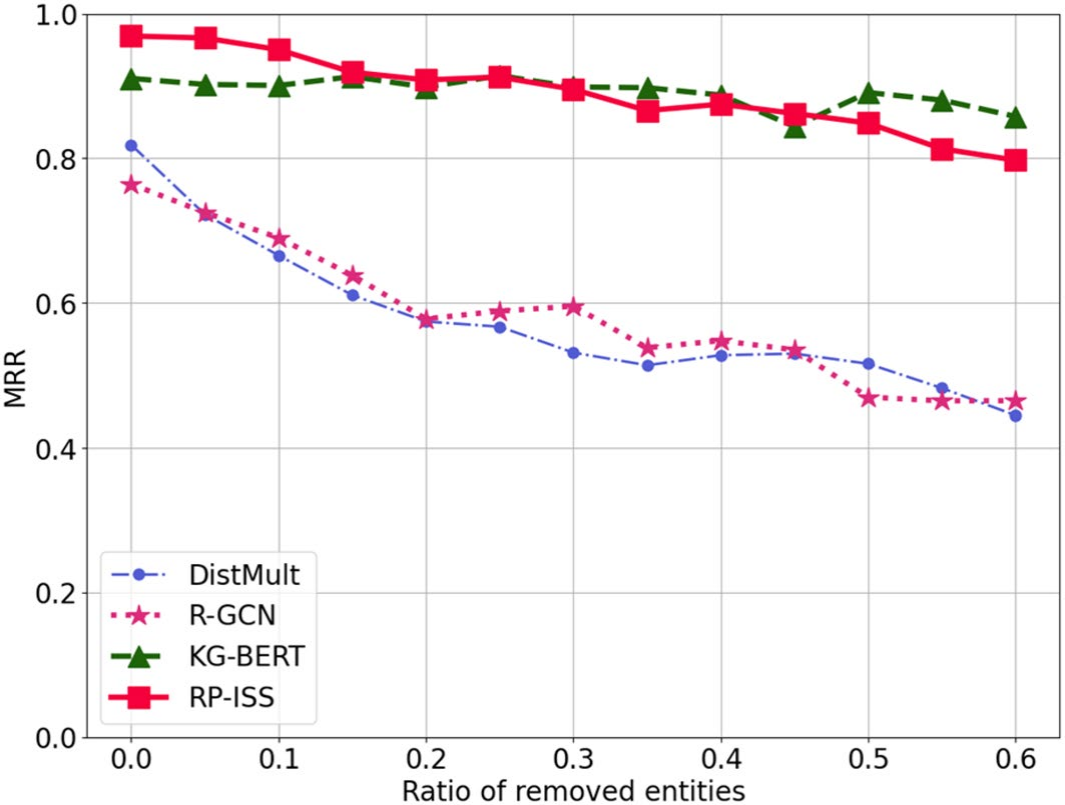
Results of RP-ISS for inductive relation prediction on WN18RR.

In Fig. 6, we compared four models' performance in inductive tasks, finding that our model and KG-BERT were effective. However, our model's performance slightly falls behind KG-BERT when more than 40% of nodes are reduced. This is likely due to differences in handling textual semantics: KG-BERT uses sequential encoding of entities for relation prediction without needing structural data, while RP-ISS uses individual entity encoding combined with structural information. KG-BERT's performance declines slowly in inductive prediction as it relies less on structural data. In contrast, RP-ISS, although also based on textual semantics, depends more on the graph's structural integrity, leading to faster performance drops as structural data decreases. We plan to further explore RP-ISS-based techniques for better handling inductive prediction challenges.

## Conclusions

Our study introduces RP-ISS, a new model for relation prediction in knowledge graphs, integrating both semantic and structural features for enhanced accuracy. RP-ISS uses BERT for semantic encoding and an edge-based relational message passing network for structural representation, with a node embedding memory bank to reduce computational costs. Tested on three classic datasets, RP-ISS showed significant improvements over models using only semantic or structural features. An ablation study confirmed the importance of both feature types. While RP-ISS surpassed GNN-based methods in inductive learning ability, it slightly lagged behind KG-BERT. Large language models like GPT-4 have introduced novel approaches to tasks related to knowledge graph completion. Pan et al.^34^ summarized some valuable ideas on leveraging LLMs to support the completion of knowledge graphs. In future research, we will further explore how to integrate the capabilities of large language models with the model proposed in this study, aiming for improved performance in completing knowledge graphs.

## Data Availability

The datasets analyzed and the code generated during the current study available from the corresponding author on reasonable request.
